# Draft Genome Sequence of “*Candidatus* Hepatoplasma crinochetorum” Ps, a Bacterial Symbiont in the Hepatopancreas of the Terrestrial Isopod *Porcellio scaber*

**DOI:** 10.1128/genomeA.00674-15

**Published:** 2015-08-13

**Authors:** Astrid Collingro, Rok Kostanjšek, Elena R. Toenshoff, Frederik Schulz, Lisa Schuster, Daryl Domann, Matthias Horn

**Affiliations:** a Department of Microbiology and Ecosystem Science, University of Vienna, Vienna, Austria; b Department of Biology, Biotechnical Faculty, University of Ljubljana, Ljubljana, Slovenia

## Abstract

“*Candidatus* Hepatoplasma crinochetorum” Ps is an extracellular symbiont residing in the hepatopancreas of the terrestrial isopod *Porcellio scaber*. Its genome is highly similar to that of the close relative “*Ca.* Hepatoplasma crinochetorum” Av from *Armadillidium vulgare*. However, instead of a clustered regularly interspaced short palindromic repeat (CRISPR)-Cas system, it encodes a type I restriction modification system.

## GENOME ANNOUNCEMENT

Terrestrial isopods (Crustacea: Isopoda) typically harbor uncultivated bacteria in their midgut glands (hepatopancreas), such as the alphaproteobacterial symbiont “*Candidatus* Hepatincola porcellionum” or the mollicute “*Candidatus* Hepatoplasma crinochetorum” (1, 2). “*Ca.* Hepatoplasma crinochetorum” is a stalk-forming bacterium, which is transmitted environmentally (3). Being intimately associated with the epithelial surface of the hepatopancreas, it seems to be beneficial to its isopod host under low-nutrient conditions (4). Previously, the genome sequence of “*Ca.* Hepatoplasma crinochetorum” strain Av, residing in the hepatopancreas of the pill-bug *Armadillidium vulgare*, was used to resolve the phylogenetic affiliation of “*Candidatus* Hepatoplasma” as a sister taxon to the *Mycoplasma hominis* group (5). Here, we report the genome sequence of “*Ca.* Hepatoplasma crinochetorum” strain Ps, an extracellular symbiont in the hepatopancreas of the rough wood louse *Porcellio scaber*.

The midgut glands of 16 animals from a laboratory population of *P. scaber* were isolated, homogenized, filtered, and DNase digested. Bacterial genomic DNA was extracted with the protocol described in reference 6 and sequenced with Illumina HiSeq 2000. Reads were mapped with the Burrows-Wheeler aligner (BWA) (7) and assembled with SPAdes 3.1 (8). The 2 contigs obtained comprised 621,166 nucleotides at 44× coverage and were annotated with Prokka-1.12beta (9), resulting in 563 predicted coding sequences (CDSs), 28 tRNAs, 1 rRNA operon, and 1 transfer-messenger RNA (tmRNA).

The genome of “*Ca.* Hepatoplasma crinochetorum” Ps shows typical features known from other *Mollicutes* genomes, including a low G+C content (23.4%) and truncated metabolic pathways. The tricarboxylic acid cycle, the pentose phosphate pathway, and the respiratory chain, with the exception of an F-type ATPase, are missing. Nucleotide synthesis and many pathways for amino acid, vitamin, and cofactor biosynthesis and cell wall synthesis are degenerated. Energy is likely produced by the introduction of various sugars into glycolysis and the arginine deiminase pathway (10). The draft genome sequence of “*Ca.* Hepatoplasma crinochetorum” Ps contains only 19 fewer CDSs than the complete genome of strain Av, and based on OrthoMCL (11), clustering the two genomes shows that they differ only in 14 and 11 genes not contained in the respective other genome. The main difference between these two highly similar “*Ca.* Hepatoplasma crinochetorum” genomes is the presence of a clustered regularly interspaced short palindromic repeat (CRISPR)-Cas system in strain Av (5) and its notable absence in strain Ps, which instead encodes a type I restriction modification system (*hsd*MRS) for defense against foreign DNA. Although this system is also found in other *Mollicutes*, like *Mycoplasma pneumoniae* (12), the type I restriction modification system of “*Ca.* Hepatoplasma crinochetorum” Ps seems to be more similar to those of members of the *Firmicutes* and may thus be of a different origin compared to other *Mollicutes*. The genome sequence of “*Ca.* Hepatoplasma crinochetorum” Ps is only the second available sequence for a member of the “*Candidatus Hepatoplasma*” genus; it should contribute to a better understanding of this group of isopod symbionts, the molecular interaction with its *P. scaber* host, and the evolution of the phylum *Mollicutes*.

### Nucleotide sequence accession numbers.

This whole-genome shotgun project has been deposited in DDBJ/ENA/GenBank under the accession numbers CWGI01000001 to CWGI01000002. The version described in this paper is the first version.
